# Undiagnosed chronic respiratory disorders in symptomatic patients with initially suspected and excluded coronary artery disease: insights from a prospective pilot study

**DOI:** 10.3389/fmed.2023.1181831

**Published:** 2023-06-15

**Authors:** Christoph Beyer, Anna Boehm, Alex Pizzini, Philipp Grubwieser, Gudrun Feuchtner, Axel Bauer, Guenter Weiss, Judith Loeffler-Ragg, Guy Friedrich, Fabian Plank

**Affiliations:** ^1^Department of Internal Medicine III – Cardiology and Angiology, Medical University of Innsbruck, Innsbruck, Austria; ^2^Department of Internal Medicine II – Infectious Diseases, Pneumology and Rheumatology, Medical University of Innsbruck, Innsbruck, Austria; ^3^Servizio Pneumologico Aziendale, Azienda Sanitaria dell’ Alto Adige, Bolzano, Italy; ^4^Department of Radiology, Medical University of Innsbruck, Innsbruck, Austria

**Keywords:** chronic respiratory disorders, chronic obstructive pulmonary disease, coronary artery disease, dyspnea, chest discomfort

## Abstract

**Background:**

Chronic respiratory diseases represent the third-leading cause of death on a global scale. Due to mutual symptoms with cardiovascular diseases and potential inappropriate attribution of symptoms, pulmonary diseases often remain undiagnosed. Therefore, we aimed to evaluate the prevalence of chronic respiratory disorders among symptomatic patients in whom suspected coronary artery disease (CAD) was ruled out.

**Methods:**

After CAD was excluded by invasive coronary angiography (ICA), 50 patients with chest pain or dyspnea were prospectively enrolled in this study. All patients underwent lung function testing, including spirometry and diffusion measurements. At baseline and the 3-month follow-up, standardized assessments of symptoms (CCS chest pain, mMRC score, CAT score) were performed.

**Results:**

Chronic respiratory disease was diagnosed in 14% of patients, with a prevalence of 6% for chronic obstructive ventilation disorders. At 3-month follow-up, patients with normal lung function tests revealed a substantial improvement in symptoms (mean mMRC 0.70 to 0.33, *p* = 0.06; median CAT 8 to 2, *p* = 0.01), while those with pulmonary findings showed non-significant alterations or unchanged symptoms (mean mMRC 1.14 to 0.71, *p* = 0.53; median CAT 6 to 6, *p* = 0.52).

**Conclusion:**

A substantial proportion of patients with an initial suspicion of coronary artery disease was diagnosed with underlying chronic respiratory diseases and exhibited persistent symptoms.

## 1. Introduction

Chronic respiratory diseases encompass a broad spectrum of various disorders affecting the airways and pulmonary structures. The global prevalence is estimated at 7.1%, with the highest rates in high-income nations (10.6%), where chronic obstructive pulmonary disease (COPD) accounts for more than half of all cases (1). As the third leading cause of death, these conditions pose a substantial global socioeconomic challenge (2).

COPD often remains underdiagnosed in the general population, which prevents patients from receiving effective treatment (3). Even though many chronic respiratory diseases cannot be cured, early diagnosis is of paramount importance to inform patients about risk factors (i.e., cigarette smoking), to allow early treatment, to prevent progression and to preserve a good quality of life. Appropriate therapy also considerably contributes to the prevention of major comorbidities (4). With a 2- to 5-fold higher risk among COPD patients, coronary artery disease (CAD) is one of the most important comorbidities (5, 6).

Moreover, patients with cardiovascular or pulmonary diseases often present with mutual initial symptoms, particularly dyspnea. In clinical practice, once coronary artery disease, the leading cause of death worldwide, as the primary diagnostic focus has been ruled out, further assessment of symptoms often remains neglected. This may impede the identification of underlying pulmonary symptoms in patients and may lead to the potential undetectability of chronic pulmonary diseases (2, 7).

Therefore, the objective of this pilot study was to investigate the prevalence of chronic respiratory diseases in symptomatic patients after CAD had been ruled out by invasive coronary angiography (ICA). As symptomatic individuals without CAD may benefit from downstream pulmonary testing, we additionally conducted a detailed assessment of symptoms in these patients which initially led to ICA and re-evaluated them after a follow-up period of 3 months.

Furthermore, this study aimed to evaluate the feasibility of our study protocol for future multicenter trials as insights from large scale clinical studies will be needed to develop effective strategies to further improve the underdiagnosis of COPD.

## 2. Materials and methods

### 2.1. Study design

This prospective pilot study was conducted as a non-randomized, open-label, single-center trial at a tertiary care university hospital.

Prior to its initiation, the local ethics committee evaluated and authorized this study. The investigation was carried out in conformity with the Declaration of Helsinki and the European Data Policy. Written informed consent was obtained from all participating patients.

The primary objective of this study was to evaluate the prevalence of chronic respiratory insufficiency in a sample of symptomatic patients after excluding CAD by ICA. The secondary objective was to examine the potential relationship between abnormal pulmonary function and prior symptom burden and characteristics, including chest discomfort and dyspnea.

**Figure 1:**
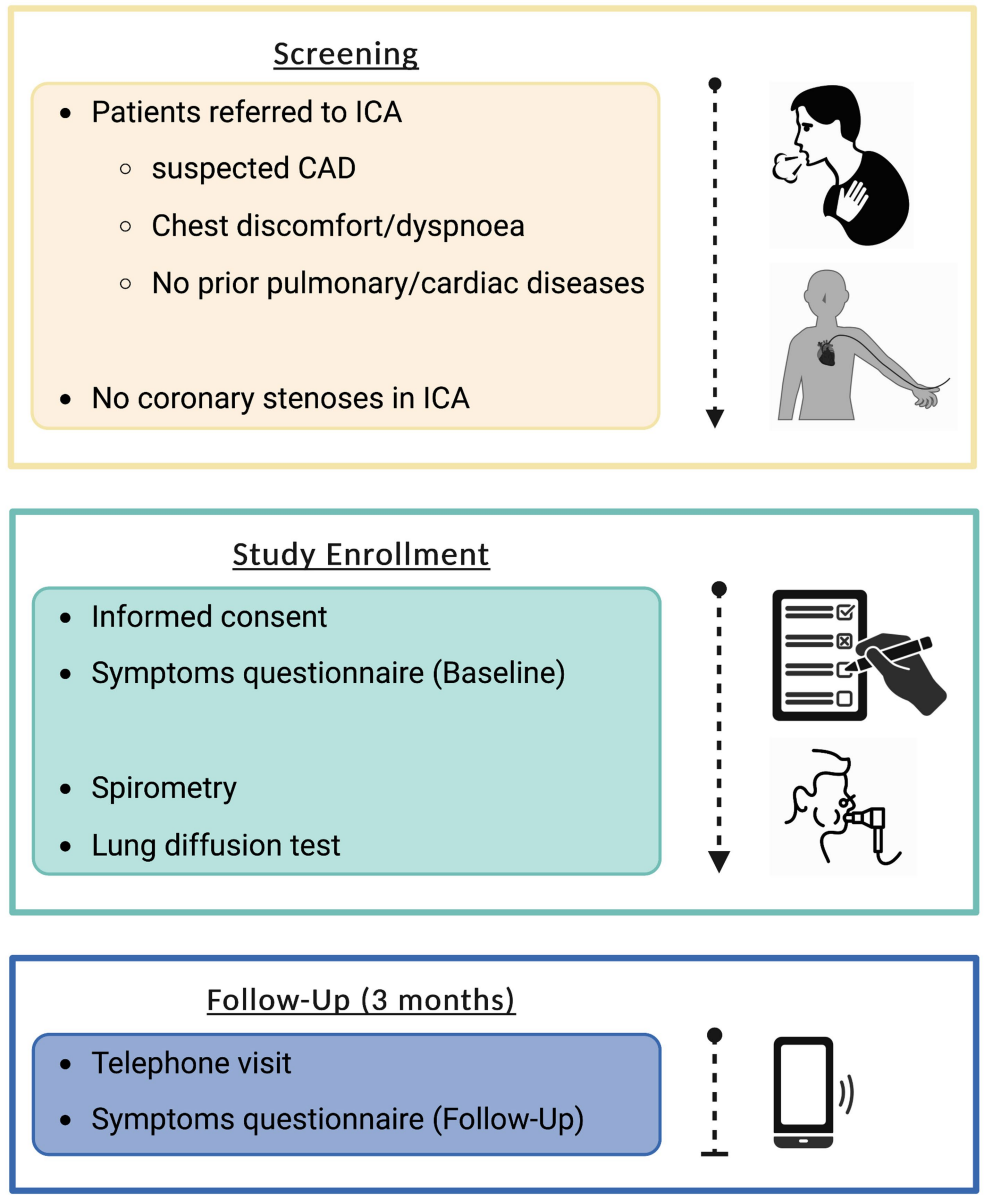
Flowchart depicting study protocol. Created with BioRender.com. CAD, coronary artery disease; ICA, invasive coronary angiography.

Figure 1 depicts the study protocol. To generate conclusive initial findings, a feasible sample size of 50 study participants was defined in this pilot study, with random patient screening based on pre-determined inclusion criteria. Following screening, patients with negative ICA findings were included in the trial after providing informed consent. A physician carried out a thorough standardized symptom evaluation the same day. The following day, spirometry (with bronchodilation in case of obstructive ventilation) and diffusion tests were performed. Every patient had a result-oriented discussion with a pulmonologist. In case of pathologic findings, patients were admitted to outpatient care providers for further tests and therapy. Follow-up was conducted after 3 months via phone to assess symptoms, medication modifications, and any adverse events.

### 2.2. Study subjects

Individuals who received invasive coronary angiography due to suspected coronary artery disease-related chest pain and/or dyspnea but revealed no evidence of coronary stenoses were recruited for this trial. Patients had to be over 18 years of age and able to provide written informed consent. This study excluded patients with either known or newly diagnosed cardiac conditions, pre-existing lung pathologies, allergies to bronchodilators, and pregnant women.

### 2.3. Symptom assessment

The standardized assessment of chest discomfort was carried out in accordance with the European Society of Cardiology’s 2019 Guidelines on Chronic Coronary Syndromes (8). Therefore, three subcategories were used: typical angina pectoris, atypical angina pectoris, and non-anginal chest pain.

The degree of dyspnea was graded from 0 to 4 using the standardized modified Medical Research Council (mMRC) severity scale (9). In addition, the COPD Assessment Test (CAT) was obtained to detect additional subjective complaints that may indicate pulmonary pathologies (10).

### 2.4. Statistical analysis

For the statistical analysis, GraphPad Prism for macOS (version 9.5.0; GraphPad Software, LLC, La Jolla, CA, United States) was used. Categorical data are presented as absolute values and percentages. Significant differences between groups were assessed using either the Chi-square test or Fisher’s exact test. Where applicable, quantitative values are reported as means ± SD or medians ± first and third quartile. Two-tailed ANOVA testing with post-hoc analyses was used to make comparisons. The Šidák correction was used to adjust for multiple comparisons. The level of significance was set at *p* < 0.05.

## 3. Results

This prospective pilot study included 50 patients, 24 (48.0%) were women. The patients’ demographics are displayed in Table 1.

**Table 1:** Demographics.

	*n* = 50
Female sex	24 (48%)
Age	64.8 ± 9.0
Body mass index	27.8 ± 4.9
Cholesterol [mg/dl]	162.4 ± 46.0
LDL [mg/dl]	90.9 ± 41.4
Creatinine [mg/dl]	0.95 ± 0.2
GFR > 60 [ml/min/1.73 m^2^]	41 (82%)
45–60	9 (18%)
Arterial hypertension	34 (68%)
under treatment	31 (62%)
Dyslipidemia	38 (76%)
under treatment	30 (60%)
Diabetes	12 (24%)
under treatment	6 (12%)
Family history of CAD	20 (40%)
Active smoking	6 (12%)
Former (>1 year)	19 (38%)
Mean packyears (range)	27.9 ± 20.1

Data are represented as *n* (%) or mean ± SD. CAD, coronary artery disease; GFR, glomerular filtration rate; LDL, low-density lipoprotein.

Seven patients (14.0%) presented with functional respiratory abnormalities, 3 (6.0%) had obstructive and 4 (8.0%) had non-obstructive pulmonary impairments (2 patients with restrictive ventilation disorder, 2 patients with pulmonary diffusion dysfunction).

**Figure 2:**
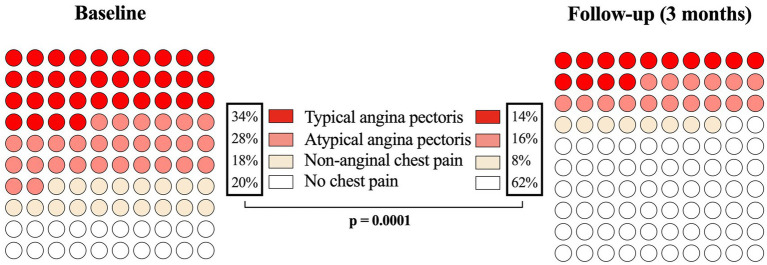
Frequencies of the various manifestations of chest discomfort in the study cohort, as classified by ESC guidelines (2019) at two timepoints: Baseline. 3-month follow-up. Data are shown as 10 × 10 dot plots and percentages.

At the three-months follow-up, the overall number of patients experiencing chest discomfort declined significantly from 40 (80.0%) at baseline to 19 (38.0%) (*p* = 0.0001). This reduction was particularly pronounced in patients who initially had typical angina pectoris, with 17 (34.0%) patients at baseline versus 7 (14.0%) at follow-up (*p* = 0.034). The number of patients with atypical angina pectoris declined from 14 (28.0%) to 8 (16.0%) (*p* = 0.23), while the number of patients with non-specific chest pain decreased from 9 (18.0%) to 4 (8.0%) (*p* = 0.07) (Figure 2). The total number of patients suffering from dyspnea also decreased significantly, from 27 (54.0%) at baseline to 13 (26.0%) at follow-up (*p* = 0.007).

**Figure 3:**
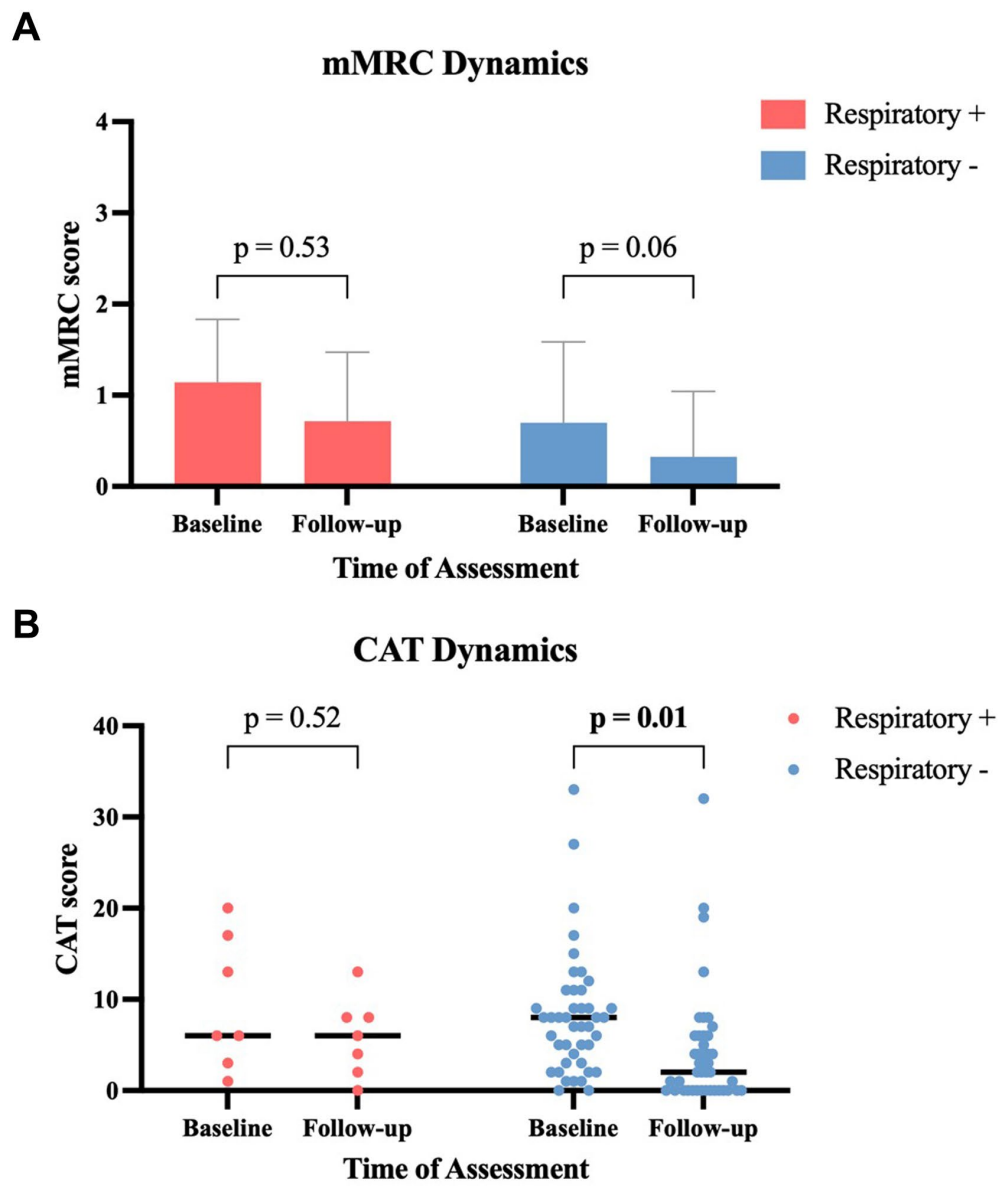
Symptom-score changes between baseline and 3-month follow-up in patients with and without abnormal respiratory tests: **(A)** modified Medical Research Council (mMRC) score (data provided as mean ± standard deviation). **(B)** COPD assessment test (CAT) score (data provided as scatter dot plot and median). CAT, COPD assessment test; mMRC, modified Medical Research Council; Respiratory+, patients with pathological respiratory tests; Respiratory−, patients with normal respiratory tests.

**Figure 4:**
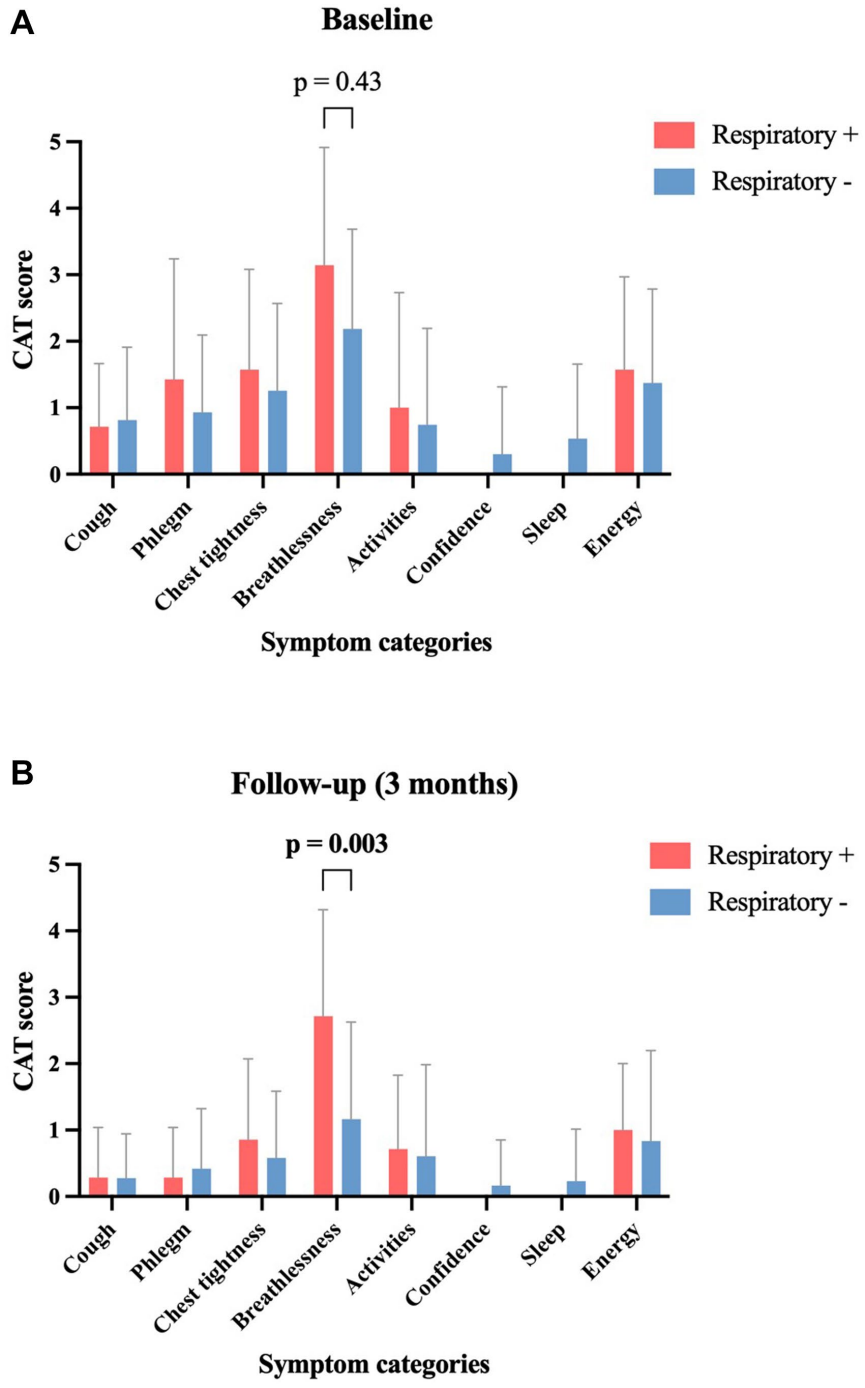
Symptom-score changes in patients with and without abnormal lung tests between baseline and 3-month follow-up, according to the 8 CAT symptom classes. Data provided as mean ± standard deviation. CAT, COPD assessment test; Respiratory+, Patients with pathological respiratory tests; Respiratory−, Patients with normal respiratory tests.

The grouped analysis of initial symptoms leading to ICA revealed that patients with subsequent abnormal respiratory function reported more cases of dyspnea (85.7% vs. 48.9%, *p* = 0.11); in contrast, they reported fewer cases of chest discomfort (57.1% vs. 83.7%, *p* = 0.13) compared to those patients without subsequent pathological pulmonary findings (Table 2). Compared to the initial symptom presentation, patients with pathological pulmonary function revealed a smaller decrease in the mean mMRC score with a decline from 1.14 to 0.71 (−37.7%, *p* = 0.53) compared to patients with normal lung function who experienced a larger decrease from 0.70 to 0.33 (−52.9%, *p* = 0.06) at the follow-up after 3 months (Figure 3A). Similar dynamics were seen regarding the CAT score after 3 months: patients with pathological respiratory findings had unaltered median CAT scores of 6 (±0; *p* = 0.52), whereas patients without pathological respiratory findings had a significant decline in median CAT score from 8 to 2 (−6; *p* = 0.01) (Figure 3B). In a subanalysis of CAT score categories at baseline, no significant difference between the two groups was found. At follow-up, patients with pathological pulmonary tests had a significantly higher mean score in the subcategory “breathlessness” compared to patients without respiratory disorders (*p* = 0.003) (Figure 4).

**Table 2:** Patient characteristics and symptoms at baseline.

	Respiratory+ (*n* = 7)	Respiratory− (*n* = 43)	value of *p*
Age	66.1 ± 10.0	64.5 ± 8.9	0.664
Female gender	3 (42.9%)	21 (48.9%)	1.000
Body mass index	29.9 ± 5.8	27.4 ± 4.7	0.205
Smoker	1 (14.3%)	5 (11.6%)	1.000
Chest pain	4 (57.1%)	36 (83.7%)	0.133
Dyspnea (mMRC ≥1)	6 (85.7%)	21 (48.9%)	0.107
CAT ≥10	3 (42.9%)	11 (25.6%)	0.384

Data are represented as *n* (%) or mean ± SD. CAT, COPD assessment test; mMRC, modified Medical Research Council; Respiratory+, patients with pathological results; Respiratory−, patients with normal results.

During the 3-month follow-up period, four patients in the group with normal pulmonary function acquired COVID-19. Symptom dynamics did not change when excluding those patients from the statistical analysis.

## 4. Discussion

This pilot study discovered a significant prevalence of abnormal respiratory function in symptomatic individuals after coronary artery disease had been ruled out by ICA, with a substantial prevalence of chronic obstructive pulmonary disease. Baseline symptom characteristics showed more dyspnea and less chest discomfort among individuals with pulmonary findings. Changes in symptoms were significantly different, with persistent dyspnea in patients with abnormal pulmonary function at follow-up. This is, to the best of our knowledge, the first prospective study to investigate the prevalence of pulmonary diseases in symptomatic patients with suspected CAD, however, in whom CAD was excluded by ICA. The results of our study support the underlying hypothesis that a considerable proportion of patients may be referred for ICA due to underlying pulmonary symptoms.

As the initial point of contact for symptomatic patients, primary care plays a crucial part in the timely diagnosis of chronic respiratory disorders. Lack of spirometry testing in primary care has been shown to be one major reason for missed diagnoses of chronic respiratory disorders (11, 12). According to the US Preventive Services Task Force, however, there are no clear indications that screening asymptomatic individuals for COPD improved their clinical outcome. Therefore, broad diagnostic testing by means of spirometry is not recommended for the general population and should be limited to individuals with risk factors or symptoms (13).

Respiratory symptoms are subjective and may vary in quality and intensity, making symptom-based pulmonary screening difficult and reliant on self-reporting by patients during medical visits (14, 15). Labonté et al. demonstrated that undiagnosed COPD patients utilized health care services to a comparable extent as diagnosed COPD patients due to exacerbation events (16). A real-world retrospective study by Jones et al. revealed that approximately 85% of COPD patients presented with respiratory symptoms to a physician in the 5 years before their diagnosis and 58% within 6 to 10 years prior to diagnosis (17). This may be in line with the results of our pilot study, which indicate that lingering symptoms after a negative ICA result might be strongly indicative of a prior undetected chronic respiratory condition.

Therefore, some studies advocate a questionnaire-based selection of patients for diagnostic spirometry as a cost-effective approach to detect chronic respiratory disease in individuals (18). Our findings support the need for enhanced screening for chronic pulmonary disorders and the feasibility of including persistent symptoms such as dyspnea and chest pain in pre-screening.

However, the reliability of questionnaire-based findings is subject to large variations among individuals, and like the quality of physician-patient dialogues, it heavily depends on the active participation of patients (19). On this basis some research suggests that handheld flow meters may be more effective than questionnaires in pre-selecting individuals for diagnostic spirometry (20).

Even if patients accurately report symptoms, general practitioners may misinterpret them, which also could substantially delay the diagnosis of lung diseases. Physicians may incorrectly attribute symptoms such as chest tightness or dyspnea to cardiovascular diseases as opposed to bronchoconstriction in asthma or COPD (21–23). Particularly physicians without pulmonary focus are prone to substantially underdiagnose COPD, while representing the first point of contact for most patients with undiagnosed pulmonary conditions (12, 24). According to a survey, most pulmonologists suggest that a lack of awareness of chronic respiratory diseases appears to be the leading cause of insufficient early diagnoses (25). However, modest educational training has been proven to significantly increase physicians’ expertise in COPD diagnosis (26). This demonstrates the yet-untapped potential of education and training in the early identification of respiratory diseases to further campaign undiagnosed chronic respiratory disorders.

While our pilot study is limited by a small sample size, our objective was to explore the prevalence of clinically confirmed chronic respiratory disorders in a highly homogeneous population. The absence of pre-existing pulmonary or cardiac conditions (i.e., heart failure, valvular heart disease) in this study population prevented potential distortion in the analysis of symptoms. The diagnostic approach of our study protocol was designed to be practical and readily accessible, hence, we restricted pulmonary function testing to spirometry and diffusion measurement. However, this approach limited the classification of potential restrictive pulmonary dysfunction to data derived from spirometry, as body plethysmography was not incorporated. The purpose of this pilot study was to assess the feasibility of the present study protocol for multicenter trials with large patient populations. Such large-scale investigations will be needed to develop effective ways to counteract pulmonary underdiagnosis in people with chest symptoms but no cardiovascular diseases.

In conclusion, following the exclusion of coronary artery disease through ICA, a relevant portion of symptomatic individuals were found to have respiratory dysfunction, with a substantial prevalence of obstructive ventilation abnormalities. Downstream pulmonary testing may be reasonable in individuals with persistent symptoms after CAD exclusion.

## Data availability statement

The raw data supporting the conclusions of this article will be made available by the authors, without undue reservation.

## Ethics statement

The studies involving human participants were reviewed and approved by Ethics committee of the Medical University of Innsbruck Approval number: 1296/2019. The patients/participants provided their written informed consent to participate in this study.

## Author contributions

CB, AnB, AP, AxB, GW, JL-R, GFr, and FP conceived and designed the study. CB, AnB, and FP collected the data. CB, AnB, AP, PG, GFe, AxB, GW, JL-R, GFr, and FP analyzed and interpreted the data, revised and approved the manuscript, and agreed to be accountable for all aspects of the work. CB and FP drafted the manuscript. All authors contributed to the article and approved the submitted version.

## Funding

This pilot study was supported by grants of the Austrian Science Fund (FWF, DOC 82 doc.fund).

## Conflict of interest

The authors declare that the research was conducted in the absence of any commercial or financial relationships that could be construed as a potential conflict of interest.

## Publisher’s note

All claims expressed in this article are solely those of the authors and do not necessarily represent those of their affiliated organizations, or those of the publisher, the editors and the reviewers. Any product that may be evaluated in this article, or claim that may be made by its manufacturer, is not guaranteed or endorsed by the publisher.
